# Exercise as a Bidirectional Regulator of Drp1: A Goldilocks Principle for Mitochondrial Adaptation in Skeletal Muscle

**DOI:** 10.3390/cells15121091

**Published:** 2026-06-16

**Authors:** Mei Ma, Jialin Li, Wentao Pang, Ziyi Zhang, Yong Zhang, Hai Bo

**Affiliations:** 1 Tianjin Key Laboratory of Exercise Physiology and Sports Medicine, Institute of Exercise and Health, Tianjin University of Sport, Tianjin 301617, China; 2 School of Physical Education, Huaibei Normal University, Huaibei 235000, China; 3 Department of Military Training Medicines, Logistics University of Chinese People’s Armed Police Force, Tianjin 300162, China

**Keywords:** Drp1, mitochondrial adaptation, skeletal muscle atrophy, ROS, exercise

## Abstract

**Highlights:**

**What are the main findings?**

- Drp1 activity follows a Goldilocks principle in skeletal muscle mitochondrial adaptation.
- ROS–Nrf2 and ROS–AMPK signaling mediate context-dependent Drp1 regulation during exercise.

**What are the implications of the main findings?**

- Exercise acts as a homeostatic regulator of Drp1 rather than a simple activator or inhibitor.
- Exercise suppresses Drp1 hyperactivation in metabolic disease while restoring deficient Drp1 activity in aging.

**Abstract:**

Dynamin-related protein 1 (Drp1) is essential for mitochondrial dynamics in skeletal muscle, particularly in regulating fission, mitophagy, and maintaining mitochondrial function. Exercise is crucial for sustaining muscle function, promoting mitochondrial adaptations that enhance energy metabolism and oxidative capacity in skeletal muscle. In this Review, we discuss the role of Drp1 in exercise-induced mitochondrial adaptations and its potential implications for skeletal muscle health. We first address the evidence that Drp1 activity must be maintained within a narrow physiological range. Both Drp1 deficiency and overabundance provoke muscle atrophy and dysfunction, establishing a Goldilocks principle for mitochondrial fission. We then examine the multi-layered post-translational modification code that governs Drp1 activity, including canonical phosphorylation, redox-sensing modifications, and the receptor selectivity model that may specify distinct fission programs. A three-stage model of exercise-induced mitochondrial adaptation is presented, describing how Drp1 activity is temporally orchestrated from acute fragmentation through short-term remodeling to long-term network optimization, and how these morphological transitions govern substrate metabolism and determine exercise performance. The pathological consequences of Drp1 dysregulation are examined in metabolic disease, where Drp1 is chronically hyperactivated, and in aging, where Drp1 activity is deficient. Finally, we analyze the ROS-Drp1 signaling axis as the mechanistic basis for the bidirectional regulation of Drp1 by exercise. Moderate exercise-induced ROS production activates Nrf2 and AMPK signaling, which suppress excessive fission in metabolic disease while restoring insufficient fission in aging, thereby moving Drp1 activity toward the physiological Goldilocks zone in both contexts. This context-dependent, bidirectional regulation distinguishes exercise from pharmacological inhibitors and identifies the ROS-Drp1 axis as a therapeutic target for conditions at opposite ends of the Drp1 activity continuum, such as sarcopenia and type 2 diabetes.

## 1. Introduction

Exercise training triggers coordinated changes in skeletal muscle gene expression, protein turnover, and organelle remodeling. Central to this adaptive response are mitochondria, whose capacity for oxidative phosphorylation determines endurance performance and metabolic health. Exercise training increases mitochondrial volume, cristae density, and respiratory chain supercomplex assembly [1,2,3], driven largely by the transcriptional coactivator PGC-1α and its downstream effector [4]. Beyond biogenesis, mitochondria are continuously remodeled by fission and fusion. These opposing processes, together termed mitochondrial dynamics, control not only mitochondrial number and morphology but also quality, distribution, and metabolic function [5,6].

Dynamin-related protein 1 (Drp1) is the principal mediator of mitochondrial fission. It is encoded by the *DNM1L* gene and functions as a cytosolic GTPase. Upon recruitment to the mitochondrial outer membrane, Drp1 assembles into ring-like oligomers that constrict and sever the organelle [5,7]. Drp1 is now recognized as a multifunctional regulator that coordinates energy supply, redox status, and transcriptional programs in skeletal muscle [8,9,10]. The phosphorylation of Drp1 at serine 616 promotes mitochondrial fission, while phosphorylation at serine 637 retains Drp1 in the cytosol and suppresses fission [11]. These two residues operate within a far more complex post-translational modification landscape that includes methylation, SUMOylation, acetylation, and S-nitrosylation, all of which tune Drp1 activity in response to cellular state [12,13,14].

The importance of Drp1 for skeletal muscle health is underscored by genetic models. Muscle-specific ablation of Drp1 in mice leads to muscle atrophy, mitochondrial swelling, disrupted calcium handling, and blocked autophagy [15,16]. Partial knockdown, which reduces Drp1 by 60–70%, causes a 40–50% loss of muscle mass, accompanied by mitochondrial dysfunction, denervation, and fibrosis [17]. However, Drp1 overexpression also causes muscle mass loss and impaired exercise performance [18]. These findings indicate that Drp1 activity must be precisely regulated within a narrow physiological window, a Goldilocks principle that is particularly critical in skeletal muscle.

Exercise presents a unique physiological challenge to this regulatory constraint. An acute bout rapidly activates Drp1 via Ser616 phosphorylation, triggering mitochondrial fragmentation [19,20]. With repeated training, both fission and fusion machineries are coordinately upregulated. Over months to years, the network adopts a hyperfused, cristae-dense mitochondrial network optimized for oxidative efficiency [1,2,21,22]. The progression from acute fragmentation through short-term remodeling to long-term optimization depends on tight control of Drp1 activity. Mitochondrial morphology, shaped by Drp1, in turn influences substrate preference. Fragmented mitochondria reduce CPT1 sensitivity to malonyl-CoA, favoring fatty acid oxidation [23], and Drp1 supports complex II assembly by enabling Sdhaf2 translocation, as demonstrated in C57BL/6J mice with muscle-specific Drp1 heterozygosity [8]. Through these effects on metabolism, Drp1 function contributes to endurance, fatigue resistance, and the adaptive response to training [20].

Exercise therefore regulates Drp1 through a bidirectional, context-dependent mechanism that adjusts fission activity toward a physiological Goldilocks zone. In metabolic diseases such as obesity and type 2 diabetes, Drp1 is chronically hyperactivated, driving excessive mitochondrial fragmentation, oxidative stress, and insulin resistance [24,25]. Exercise suppresses this pathological overactivation and restores network integrity [25,26]. In aging skeletal muscle, Drp1 expression and activity are deficient, resulting in impaired fission capacity, impaired mitophagy, and accumulation of damaged organelles [27,28,29]. Exercise restores Drp1 function in this context, in part through ROS-mediated activation of Nrf2 and subsequent Drp1 deubiquitination [30]. Reactive oxygen species serve as the primary signal in both directions. Moderate exercise-induced ROS production activates adaptive signaling cascades that tune Drp1 activity, whereas chronically elevated ROS in metabolic disease or insufficient ROS signaling in aging drive Drp1 activity away from the optimal range. Understanding the ROS-Drp1 signaling axis and its modulation by exercise thus provides a mechanistic foundation for the Goldilocks principle that governs mitochondrial fission in skeletal muscle.

This review first establishes that Drp1 is the principal regulator of mitochondrial dynamics in skeletal muscle, and that its activity must be confined within a narrow physiological range. It then examines how a multi-layered post-translational modification code and context-dependent receptor engagement enable precise control of Drp1. A three-stage model is next presented to frame how exercise temporally orchestrates Drp1 activity from acute fragmentation through long-term network optimization, linking these morphological transitions to metabolic adaptation and performance. The opposing patterns of Drp1 dysregulation in metabolic disease and aging are then compared. Finally, the ROS-Drp1 signaling axis is discussed as the mechanistic basis for bidirectional regulation by exercise.

A literature search was conducted in PubMed and Web of Science for articles published through March 2026. Search terms included combinations of “Drp1,” “mitochondrial dynamics,” “mitochondrial fission,” “skeletal muscle,” “exercise,” “aging,” “sarcopenia,” “obesity,” “type 2 diabetes,” and “reactive oxygen species.” Original research articles and authoritative reviews in peer-reviewed English-language journals were prioritized, and reference lists of retrieved articles were screened for additional studies. Articles were selected based on their relevance to the topics of each section and the strength of mechanistic evidence linking Drp1 regulation to skeletal muscle physiology or pathology.

## 2. Mitochondrial Dynamics in Skeletal Muscle: A Drp1-Dependent Balance

### 2.1. Mitochondrial Dynamics in Skeletal Muscle: A Delicate Balance

Skeletal muscle mitochondria are not static organelles but continuously undergo fission and fusion to adapt their morphology to the cellular environment. Fusion is mediated by mitofusins 1 and 2 (MFN1, MFN2) on the outer membrane and optic atrophy protein 1 (OPA1) on the inner membrane, whereas the primary fission effector is dynamin-related protein 1 (Drp1), a cytosolic GTPase that is recruited to the mitochondrial surface to constrict and cleave the organelle [5,6]. The balance between these opposing processes determines mitochondrial number, morphology, distribution, and quality. Deletion of *Mfn1* and *Mfn2* in skeletal muscle leads to severe mitochondrial myopathy with muscle mass reduction, exercise intolerance, and lactic acidosis [31]. Muscle-specific ablation of *Opa1* in C57BL/6J mice causes mitochondrial dysfunction, oxidative stress, inflammation and leads to loss of muscle mass and strength [32]. These phenotypes establish that fusion is essential for muscle health. Fission is equally indispensable, and its principal executor, Drp1, must be regulated within a narrow physiological range.

### 2.2. Drp1 as an Indispensable Regulator of the Balance

Complete muscle-specific ablation of Drp1 in C57BL/6J male mice aged 8 to 10 weeks results in a severe myopathy with muscle atrophy, weakness, fiber degeneration and regeneration, accompanied by mitochondrial swelling, disrupted Ca^2+^ handling and blocked autophagy [16]. This severe myopathic phenotype is not restricted to the complete absence of the protein. Dulac et al. showed that adeno-associated virus-mediated Drp1 knockdown in C57BL/6J male mice aged 8 weeks, which reduces Drp1 expression by roughly 60–70%, is sufficient to cause a 40–50% loss of muscle mass within four months, together with mitochondrial dysfunction, impaired autophagy, denervation and fibrosis [17]. Thus, even a partial reduction in Drp1 levels leads to a marked loss of muscle homeostasis. Similarly, Drp1 excess is also detrimental. Muscle-specific Drp1 overexpression in FVB/N mice impairs skeletal muscle growth through translational attenuation, leading to muscle mass loss [18]. The fact that both Drp1 deficiency and Drp1 overabundance provoke a degenerative muscle phenotype establishes a classic Goldilocks principle for mitochondrial fission: too little activity is pathogenic, and too much is as well.

While the observation that both extremes are detrimental defines the permissible range of Drp1 activity, it remains unclear whether the underlying pathology is caused by the loss of fission itself or by the resulting imbalance between fission and fusion. Epistasis experiments address this question. Mice lacking the fusion protein OPA1 develop mitochondrial dysfunction and sarcopenia. Remarkably, simultaneous deletion of Drp1 partially rescues these abnormalities and prevents sarcopenia [33]. Conversely, in Drp1-knockout C2C12 myotubes, inhibiting the fusion factors MFN1/2 or OPA1, either pharmacologically or genetically, partially restores fast-twitch myosin heavy chain expression and normalizes mitochondrial morphology [34]. These reciprocal rescue experiments indicate the primary defect is not the loss of fission itself, but the disruption of the balance between fission and fusion. Both deficiency and overabundance of Drp1 are therefore pathogenic because each displaces the fission–fusion equilibrium outside a narrow functional operating range. Consequently, Drp1 is best understood not as a constitutively required fission engine, but as the principal regulator of mitochondrial fission whose activity must be precisely controlled to maintain the fission–fusion equilibrium within a functional operating range.

### 2.3. Drp1-Dependent Mitochondrial Dynamics Determine Muscle Fiber Type Identity

Drp1-regulated mitochondrial dynamics also contribute to the specification of muscle fiber type. Oxidative fibers (type I and IIA) contain elongated, highly interconnected mitochondrial networks supported by active fusion, whereas glycolytic fibers (type IIX and IIB) display more fragmented mitochondrial morphology [31]. This correlation suggests that mitochondrial architecture is not a passive consequence of contractile activity but an active participant in the specification of fiber identity.

**Figure 1:**
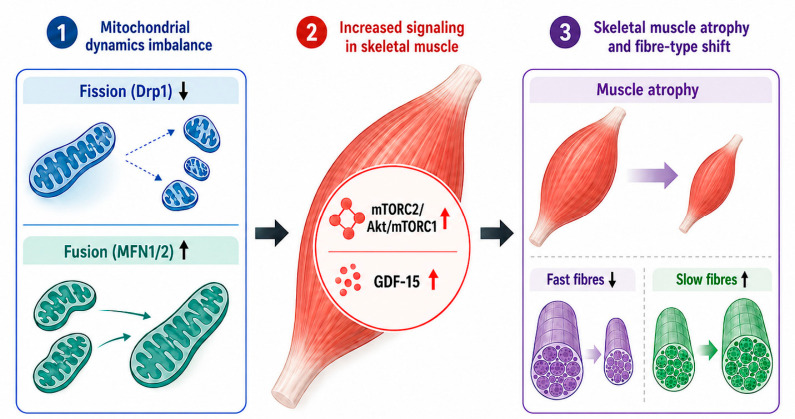
Dysregulation of mitochondrial dynamics reduces fast-twitch fiber differentiation. Deletion of Drp1 in skeletal muscle disrupts the balance between mitochondrial fission and fusion, leading to upregulation of mTORC2 and activation of the Akt/mTORC1 signaling pathway, accompanied by increased expression of GDF-15. These changes ultimately result in the loss of fast-twitch muscle fibers and skeletal muscle atrophy. Black arrows indicate changes in mitochondrial dynamics and muscle fiber types; red arrows indicate increased signaling pathways or factors; thick blue arrows indicate the proposed pathological progression. Created with BioRender.com under a publication license.

Direct evidence for this concept came from a developmental model of muscle hypertrophy. Yasuda et al. reported that Drp1 deletion in this context causes a selective loss of fast-twitch fibers, coupled with upregulation of mTORC2–Akt–mTORC1 signaling and increased expression of growth differentiation factor 15 (GDF-15) [34]. The causal link was further supported by a counterintuitive rescue experiment: in Drp1-null myotubes, acute inhibition of the fusion machinery, by targeting either MFN1/2 or OPA1, partially but significantly restored *Myh1* (fast myosin heavy chain) expression and re-established a more normal mitochondrial morphology [34]. In other words, forcibly reducing fusion in a fission-deficient background alleviated the differentiation block. The pathological state that compromised fast-fiber formation was therefore unopposed fusion rather than the absence of fission. This chronically hyperfused state, in which the irreversible loss of fission capacity skews the network permanently toward excessive elongation, is distinct from the transient stress-induced mitochondrial hyperfusion that can serve a protective function under acute conditions (Figure 1).

These observations point to a key principle. Drp1-mediated fission serves as a critical determinant of muscle fiber type identity, and its activity must remain within a narrow permissive window. When Drp1 activity drops below this window, the mitochondrial network becomes chronically hyperfused, and the fast-fiber differentiation program fails. Conversely, when Drp1 is hyperactivated, as seen in certain metabolic disorders, excessive fragmentation damages the network and impairs muscle function. Pathology at both extremes means that any intervention aimed at restoring muscle health must be able to suppress Drp1 activity when it is excessive and restore it when it is deficient. The molecular regulation of Drp1 is well suited to meet this requirement.

## 3. Drp1 Structure and Regulation

### 3.1. Domain Organization of Drp1

**Figure 2:**
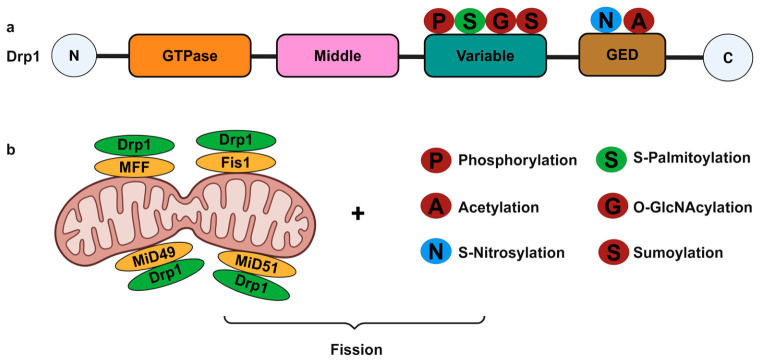
Structural components and functions of Drp1. (**a**). Schematic representation of the Drp1 structure. From the N-terminus to the C-terminus, Drp1 is composed of a GTPase domain, a middle domain, a variable domain, and a GTPase effector domain. These domains contain multiple post-translational modification (PTM) sites. (**b**). Drp1 is recruited to the mitochondrial outer membrane by adaptor proteins MiD49, MiD51, MFF, and Fis1. At mitochondrial fission sites, Drp1 assembles into ring-like structures that constrict the membrane, leading to the division of the mitochondrion into two daughter mitochondria. Created with BioRender.com under a publication license.

Drp1 comprises several structural domains, including the GTPase domain, the middle domain, the variable domain, and the GTPase effector domain (GED). Multiple post-translational modification (PTM) sites reside within the variable domain and the GED. Drp1-mediated mitochondrial fission occurs through a stepwise process. Drp1 translocates from the cytoplasm to the outer mitochondrial membrane (OMM), where it assembles into oligomeric complexes. GTPase activity is then activated, driving membrane remodeling and constriction, ultimately resulting in mitochondrial fission and the generation of daughter mitochondria [5]. Four OMM receptors recruit Drp1 from the cytoplasm: mitochondrial dynamics proteins 49 and 51 (MiD49 and MiD51), mitochondrial fission factor (MFF), and mitochondrial fission 1 protein (Fis1) (Figure 2).

In addition to its role in mitochondrial fission, Drp1 participates in mitophagy, apoptosis, calcium handling, and peroxisomal fission [5,35,36]. In skeletal muscle, Drp1 also supports myogenic differentiation. During early myogenic differentiation, Drp1-mediated mitochondrial remodeling and a moderate elevation in mitochondrial ROS levels facilitate the metabolic reprogramming required for initiating differentiation. Loss of Drp1 markedly impairs both regenerative capacity and differentiation potential of muscle cells [37]. These diverse functions underscore that the loss of Drp1 regulation can lead to severe physiological dysfunction. The multiple outputs of Drp1 depend critically on its precise regulation.

### 3.2. Canonical Post-Translational Modifications

The activity of Drp1 is orchestrated by an array of post-translational modifications (PTMs) that together constitute a dynamic “PTM code”. Rather than acting as simple on/off switches [12], these modifications function collectively through coordinated allosteric interactions to fine-tune Drp1’s oligomeric state, subcellular localization, and interactions with mitochondrial receptors [13]. Among the known PTMs, phosphorylation at Ser616 and Ser637 is considered a central mechanism regulating Drp1 function. It has been shown that the cyclin-dependent kinase CDK1/cyclin B complex phosphorylates Drp1 at Ser616, thereby promoting its oligomerization and enhancing mitochondrial fission activity [38]. Conversely, Ser637 can be phosphorylated by protein kinase A (PKA), thereby retaining Drp1 in the cytoplasm and suppressing its fission-promoting function [39]. Thus, these two residues represent key regulatory nodes that promote and inhibit mitochondrial fission, respectively. Under physiological conditions of exercise, these two phosphorylation events exhibit marked time-dependent changes. In human skeletal muscle, acute resistance exercise has also been demonstrated to significantly increase Drp1 Ser616 phosphorylation, accompanied by reductions in the mitophagy-related proteins Parkin and BNIP3L/NIX. These findings indicate that mitochondrial fission is activated before autophagic clearance occurs [40]. Notably, although AMPK is a key exercise-induced energy sensor, current evidence suggests that it may not be the only upstream regulator of Drp1 phosphorylation. This implies that Drp1 phosphorylation may be cooperatively mediated by multiple signaling pathways [41]. In addition to phosphorylation, the PTM repertoire of Drp1 includes methylation, SUMOylation, ISGylation, acetylation, and S-nitrosylation. Among these modifications, the arginine methyltransferase CARM1 has been shown to methylate Drp1 at R403 and R634 under oxidative stress conditions [14]. This methylation enhances Drp1 binding to the fission factor MFF, promoting its recruitment to mitochondria, which in turn drives fission, reduces oxygen consumption, and increases ROS production. The elevated ROS further promotes CARM1 translocation to the cytoplasm, creating a positive feedback loop that amplifies Drp1 methylation and accelerates cellular senescence. In aged skeletal muscle, where basal Drp1 expression is already low, this CARM1-Drp1 methylation axis may underlie how ROS accumulation exacerbates mitochondrial fragmentation and functional decline. This feedforward mechanism exemplifies how a single PTM can sustain pathological fragmentation. Phosphorylation and methylation thus represent key, but not exclusive, components of the PTM network that precisely controls Drp1 activity.

### 3.3. Redox-Sensing Post-Translational Modifications of Drp1

In addition to the modifications described above, Drp1 is also regulated by a group of redox-sensitive post-translational modifications mediated by reactive oxygen and nitrogen species. These modifications enable the mitochondrial fission machinery to sense and respond to the cellular redox environment, providing a potential coupling mechanism between metabolic state and mitochondrial dynamics.

S-Nitrosylation is one of the most well-established redox modifications of Drp1. Under nitrosative stress, Drp1 undergoes S-nitrosylation at cysteine 644. This modification promotes Drp1 dimerization, enhances its GTPase activity, and facilitates its recruitment to the mitochondrial outer membrane [42,43]. In neuronal models, S-nitrosylation of Drp1 has been linked to β-amyloid-induced mitochondrial fragmentation and synaptic damage [42]. Although the role of Drp1 S-nitrosylation in skeletal muscle remains largely unexplored, this modification illustrates a general principle, namely that redox-dependent modifications can directly alter Drp1’s oligomeric state and membrane affinity without requiring upstream kinase signaling.

Oxidative stress also modulates Drp1 through kinase-dependent pathways. For example, activated protein kinase C delta (PKCδ) directly phosphorylates Drp1 under oxidative conditions. The resulting Drp1-PKCδ complex translocates to the mitochondrial outer membrane, where Drp1 engages Fis1 to promote fission [44]. This pathway represents a direct link from redox stress to fission via specific kinase-substrate interaction. Separately, hyperglycemia-induced ROS can activate TRPM2 channels, triggering calcium influx, lysosomal permeabilization, and zinc release. The subsequent rise in mitochondrial zinc enhances Drp1 recruitment and stimulates fission [45]. This indirect route illustrates how redox stress can initiate ion signaling cascades that ultimately converge on the mitochondrial fission machinery.

Acetylation serves as another redox-responsive mechanism regulating Drp1. In dopaminergic neurons under oxidative stress, elevated Drp1 acetylation stimulates its oligomerization, driving mitochondrial fragmentation and dysfunction. This modification is reversed by the NAD^+^-dependent deacetylase SIRT3, which deacetylates Drp1 at lysine 711 to restore mitochondrial function [46]. Thus, this reversible acetylation-deacetylation cycle directly couples Drp1 activity to the cellular NAD^+^/NADH ratio, a central indicator of metabolic and redox state.

The ubiquitin-proteasome system also links redox state to Drp1 regulation. The cytosolic E3 ligase Parkin ubiquitinates Drp1, targeting it for proteasomal degradation. Under nitrosative stress, however, Parkin itself undergoes S-nitrosylation, which inactivates its ligase function. This impairs Drp1 degradation, leading to Drp1 accumulation and increased mitochondrial fission [47]. Thus, the fission–fusion balance can be shifted by redox modification of a Drp1 regulator, rather than of Drp1 itself.

Collectively, these redox-sensitive modifications extend the allosteric model of Drp1 regulation. They may enable the fission machinery to sense cellular redox status and integrate this information with other signaling inputs, including energy status and mechanical cues, thereby contributing to the regulation of fission activity.

### 3.4. Drp1 Receptor Selectivity: A Context-Dependent Model

The functional output of this multi-layered PTM code is strongly influenced by the membrane receptor that recruits Drp1 to the mitochondrial surface. Four distinct Drp1 receptors exist on the mitochondrial outer membrane: MiD49, MiD51, MFF, and Fis1. Whether these receptors serve redundant roles or instead specify distinct fission programs is unresolved.

In cardiac ischemia–reperfusion injury, MiD51 has been identified as a key mediator of pathological Drp1 recruitment to mitochondria. Ischemia-reperfusion upregulates MiD51 expression, and this upregulation contributes to excessive mitochondrial fragmentation and myocardial injury [48]. In contrast, under basal metabolic conditions, MFF acts as the predominant receptor for constitutive Drp1 recruitment [49]. These observations suggest that receptor usage is not fixed but dynamically regulated by cellular state.

In skeletal muscle, direct evidence for receptor-specific functions is emerging. Voos et al. [50] demonstrated that ankyrin B, a scaffold protein, assembles a ternary complex with Drp1 and MFF that promotes Drp1 oligomerization and mitochondrial recruitment. Disrupting this complex impairs fatty acid oxidation and reduces exercise endurance, suggesting a specific MFF-dependent fission pathway necessary for metabolic adaptation. In contrast, the MiD49/51 axis operates in different contexts. In older adults, MiD49 protein levels are elevated in type II muscle fibers, possibly as a compensatory response to age-related mitochondrial dysfunction. Notably, high-intensity exercise training attenuates this age-associated increase [51]. Thus, while both responsive to exercise and aging, the MiD49/51 and MFF axes appear to regulate distinct aspects of mitochondrial plasticity.

Fis1, a highly evolutionarily conserved Drp1 receptor, adds further complexity. Structural studies reveal that Fis1 can directly interact with Drp1 through its N-terminal arm and a conserved surface motif containing the SKY insert [52]. Mutations in these regions yield opposing effects on mitochondrial morphology, ranging from marked elongation to severe fragmentation, suggesting that Fis1-Drp1 interaction is tightly regulated and that its functional output depends on the precise mode of engagement.

Collectively, these findings support a model in which distinct Drp1 receptors are preferentially engaged in different contexts. In skeletal muscle, MFF may primarily mediate metabolic adaptation by promoting fission in response to exercise-induced energy demand. In contrast, the MiD49/51 axis may regulate stress- and age-related mitochondrial remodeling, while the evolutionarily conserved Fis1 could contribute to processes like atrophy or differentiation. Notably, this working model relies on correlative data and isolated studies, lacking direct causal validation. Critical tests will require skeletal muscle-specific conditional knockout models for each receptor, coupled with phenotypic characterization across endurance, resistance, and high-intensity exercise regimens. If validated, distinct receptor-dependent fission programs would be identified. These programs would then offer a molecular basis for the bidirectional regulation of Drp1 by exercise. The same upstream signal could produce opposite functional outcomes depending on whether the basal state is one of pathological hyperactivation or deficiency.

## 4. Drp1 in Exercise: From Adaptive Mechanisms to Performance Outcomes

### 4.1. Dynamic Regulation of Drp1 by Acute and Chronic Exercise

**Figure 3:**
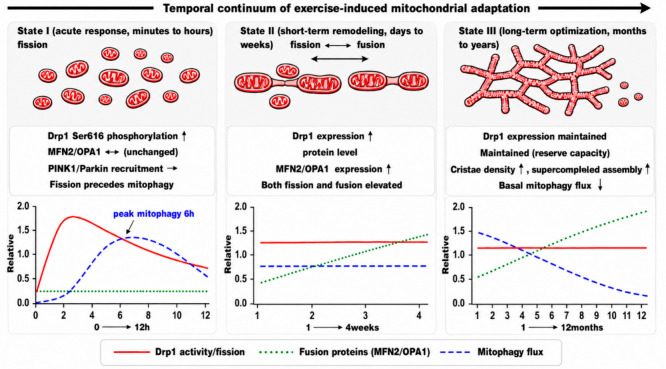
Three-stage dynamic model of mitochondrial network remodeling during exercise training. Stage I (acute response, minutes to hours): Drp1 Ser616 phosphorylation rises rapidly. This triggers transient mitochondrial fragmentation, preparing damaged segments for clearance. Mitophagy becomes prominent with a delay (~6 h post-exercise). Fusion protein levels remain largely unchanged during this period. Stage II (short-term remodeling, days to weeks): Both fission and fusion proteins are upregulated. Drp1 protein levels increase together with MFN2 and OPA1, establishing a dynamic equilibrium between the two processes. Stage III (long-term optimization, months to years): The mitochondrial network becomes hyperfused and highly interconnected, with increased cristae density and supercomplex assembly. Basal mitophagy flux declines, but fission capacity is retained through maintained Drp1 expression. The three stages form a continuum that extends from acute quality control to long-term structural and functional optimization. Upward and downward arrows denote increases and decreases, respectively; “↔” denotes unchanged levels; double-headed arrows indicate dynamic balance, and horizontal arrows indicate temporal progression.

Mitochondrial dynamics during exercise unfold dynamically across distinct temporal phases. Accumulating evidence supports a three-stage model that describes how Drp1 activity and mitochondrial network architecture evolve from the initial exercise bout through long-term training. These stages represent a continuum of molecular and structural changes that collectively contribute to skeletal muscle adaptation to exercise (Figure 3).

The earliest response to an exercise bout is a rapid and transient surge in mitochondrial fission. Previously, we found that an acute bout of prolonged treadmill running in rats rapidly altered the expression of mitochondrial fusion and fission proteins in skeletal muscle, suppressing MFN1/2 while upregulating Fis1 in a duration-dependent manner [53]. Within minutes to hours of exercise onset, Drp1 Ser616 phosphorylation increases markedly in both rodent and human skeletal muscle and is associated with a shift in mitochondrial network configuration toward fragmentation [19,20]. This phosphorylation does not appear to be solely dependent on AMPK, as previously assumed, and alternative upstream kinases may mediate the acute fission response [19,41]. The functional significance of this acute fragmentation extends beyond structural remodeling. Fragmented mitochondria are thought to be more readily trafficked along the cytoskeletal network, a mechanism that may facilitate their delivery to subcellular regions with elevated energy demands in skeletal muscle [54,55]. In mouse skeletal muscle, MitoTimer imaging has shown that mitochondrial oxidative stress peaks immediately after exercise, followed by a progressive increase in mitophagy that becomes significant approximately six hours post-exercise, as indicated by the co-localization of MitoTimer-positive puncta with lysosomal markers [19]. Importantly, the execution of mitophagy appears to be temporally dissociated from the initial fission signal. In human skeletal muscle, acute endurance exercise increases Drp1 Ser616 phosphorylation and upregulates the mRNA expression of fission-related genes, yet markers of active mitophagy remain unchanged immediately after exercise and up to one hour into recovery [56]. Similarly, a single bout of resistance exercise increases Drp1 Ser616 phosphorylation while reducing Parkin and BNIP3L/NIX protein abundance, despite ultrastructural evidence of increased mitophagosome-like structures [40].

These findings support a model in which acute exercise-induced fission may generate a pool of fragmented mitochondria that are subsequently marked for clearance, with the execution of mitophagy delayed for several hours into the recovery period. A recent computational systems model has formalized this concept, predicting that exercise induces Drp1-driven mitochondrial fission during the active phase, followed by MFN1/2- and OPA1-mediated refusion as energy stress subsides, a sequence in which fission may identify and separate damaged mitochondrial segments for degradation, whereas fusion subsequently helps reassemble the remaining healthy network [57].

As exercise bouts are repeated over days to weeks, the mitochondrial network enters a phase of dynamic remodeling in which the fission and fusion machineries are concurrently upregulated. This stage represents a transition from an acute response to a sustained adaptive program. In trained mouse skeletal muscle, the levels of mitochondrial fusion proteins OPA1 and MFN2, as well as the fission regulator Drp1, are significantly increased compared with sedentary controls [21]. Similarly, in rats subjected to endurance exercise, OPA1 and MFN2 protein expression is significantly elevated, whereas their expression is notably decreased in muscle subjected to long-term disuse [58]. In healthy trained adults, maximal oxygen uptake and mitochondrial respiratory function are significantly higher than in untrained healthy adults, with a notable rise in both *MFN2* and *DNM1L* mRNA expression [59]. These findings indicate that chronic contractile activity promotes coordinated upregulation of both the fission and fusion components of the mitochondrial dynamic machinery, rather than a simple shift toward fusion.

The functional importance of this coordinated upregulation becomes evident when Drp1 activity is partially compromised. Mice with muscle-specific Drp1 heterozygosity exhibit reduced endurance capacity and impaired running performance, together with attenuated adaptations to exercise training, despite retaining partial Drp1 expression [20]. These data suggest that the increase in Drp1 observed during short-term training is not merely incidental but may be required to support the enhanced mitochondrial remodeling and turnover associated with training-induced mitochondrial biogenesis. Exercise training is thought to enhance mitochondrial quality and quantity through the coordinated regulation of mitochondrial biogenesis, fission, fusion, and mitophagy [60]. The relative balance between fission and fusion during this adaptive phase may also depend on exercise modality. A recent computational analysis predicted that endurance exercise induces the most pronounced and sustained remodeling of mitochondrial dynamics, whereas sprint exercise produces sharp but transient fission–fusion cycles and resistance exercise elicits comparatively limited remodeling [57]. In the same model, global sensitivity analysis identified AMPK/PGC-1α signaling as a major driver of MFN1/2-mediated fusion, while ROS- and AMPK-dependent signaling through MFF/Drp1 was predicted to promote mitochondrial fission [57].

With sustained training over months to years, the mitochondrial network undergoes a long-term adaptive transformation characterized by enhanced connectivity, increased cristae density, and attenuation of basal mitophagy flux. Mitochondrial volume density increases by up to 40 percent following endurance training [61], and mitochondrial density in skeletal muscle has been shown to rise by approximately 50 to 100 percent after six weeks of continuous exercise training [62]. The initial increase in the tubular-reticular mitochondrial network appears to rely on an increase in cross-sectional area, and once this area reaches a critical threshold, further increases result from longitudinal growth [63]. Cristae density is significantly higher in trained individuals [64] and is a better predictor of maximal oxygen uptake than mitochondrial volume alone [2]. Endurance exercise training enhances the formation of mitochondrial supercomplexes in skeletal muscle, significantly improving mitochondrial respiratory function [3,64] and reducing oxidative damage [65].

Subsarcolemmal and intermyofibrillar mitochondria exhibit distinct adaptive dynamics during prolonged training. Intermyofibrillar mitochondria increase progressively throughout the training period and play a dominant role in enhancing oxidative phosphorylation capacity and meeting the energetic demands of muscle contraction [66]. Subsarcolemmal mitochondria show a more pronounced increase in content during the later stages of training, which may be associated with an increased proportion of type I muscle fibers, and their citrate synthase activity increases significantly following training, indicating enhanced oxidative capacity particularly in fatty acid oxidation pathways [66,67]. At the protein level, chronic resistance training in older adults aged 65 to 80 years significantly increases the levels of MFN1, MFN2, and OPA1, while Drp1 protein expression is likewise elevated [22]. After ten weeks of training, no significant changes are observed in mitophagy markers such as PTEN-induced kinase 1 (PINK1) and Parkin RBR E3 ubiquitin protein ligase (Parkin), suggesting that the training-induced increase in mitochondrial content may be accompanied by a lower basal rate of mitochondrial degradation, possibly reflecting an overall improvement in organelle quality [22,68]. The long-term adaptive state should not be interpreted as a fusion-only endpoint. The sustained elevation of Drp1 protein levels in trained muscle indicates that fission capacity is not lost but is preserved as a reserve mechanism. Long-term training thus produces a dynamic equilibrium between mitochondrial fission and fusion characterized by a fusion-biased network architecture while retaining sufficient fission potential to respond rapidly to subsequent metabolic challenges or acute injury.

The three-stage model thus describes the temporal sequence through which exercise drives Drp1 activity and mitochondrial network architecture toward the physiological Goldilocks zone when the starting point lies within the normal range. This temporal framework, however, does not address a distinct question, namely when the basal state is already displaced from the physiological range before exercise begins. In metabolic diseases, Drp1 is chronically overactivated, and the network is already excessively fragmented. In aging skeletal muscle, Drp1 expression and fission capacity are already deficient. The model of exercise-induced Drp1 regulation under pathological conditions follows a different logic from the three-stage adaptive sequence. Here, exercise does not operate along a fixed temporal sequence but in a corrective, bidirectional manner dictated by the underlying pathology. It suppresses excessive fission in metabolic disease and restores deficient fission in aging. This constitutes a complementary, state-dependent correction model. One framework describes the temporal dynamics of normal adaptation. The other addresses the correction of pathological deviations. Both ultimately converge on the same physiological optimum, the Goldilocks zone for mitochondrial fission.

### 4.2. Metabolic Consequences of Drp1-Mediated Mitochondrial Remodeling

The morphological transitions described in the three-stage model are associated with measurable metabolic consequences, because Drp1, by governing mitochondrial network configuration, can influence substrate preference and oxidative capacity. Ngo et al. [23] demonstrated that fragmented mitochondria display reduced sensitivity of CPT1 to malonyl-CoA inhibition in L6 myotubes. This desensitization enhances long-chain fatty acid oxidation. In the context of acute exercise, when Drp1 Ser616 phosphorylation rises rapidly and the mitochondrial network fragments, this mechanism may serve to accelerate lipid utilization at a time of heightened energy demand. Conversely, when Drp1 is absent and the network becomes excessively fused, CPT1 sensitivity to malonyl-CoA is restored, fatty acid oxidation capacity declines, and muscle accumulates lipid [69]. These findings link Drp1 activity, mitochondrial morphology, and metabolic flux. Direct extrapolation of these findings to exercise requires caution, however, because the Ngo study examined two extreme morphological states in vitro. Acute exercise-induced fragmentation is modest and transient. Whether it alters CPT1 sensitivity to a physiologically meaningful degree in vivo remains an open question. Furthermore, the rate of ATP turnover during contraction is the dominant determinant of metabolic flux in exercising muscle. The contribution of CPT1 sensitivity to overall substrate preference must therefore be evaluated within this demand-driven framework.

Drp1 loss also produces metabolic consequences beyond fatty acid handling. In male mice with muscle-specific Drp1 knockdown, Zhou et al. identified a specific mechanism through which Drp1 supports oxidative phosphorylation. Drp1 is required for the mitochondrial translocation of Sdhaf2, a chaperone essential for the assembly and activity of electron transport chain complex II [8]. When Drp1 is absent, Sdhaf2 translocation is impaired, complex II activity declines, and ATP generation is reduced. Male C57BL/6J mice with muscle-specific Drp1 heterozygosity show decreased oxidative metabolic capacity, increased lactate accumulation, increased lipid deposition, and diminished insulin sensitivity, a metabolic profile that reflects impaired substrate oxidation [20]. Ribas et al. [69] reported that this metabolic regulatory role of Drp1 is partly sex-dependent, based on studies in 24-week-old muscle-specific ERα knockout C57BL/6J mice. Skeletal muscle estrogen receptor alpha signaling may converge on Drp1 to maintain mitochondrial function and metabolic homeostasis in females.

Pharmacological experiments using the Drp1 inhibitor Mdivi-1 reveal an apparent paradox. In engineered skeletal muscle tissue derived from C2C12 myoblasts, Mdivi-1 treatment increased mitochondrial volume, maximal respiratory capacity, and contractile stress, and also elongated sarcomere length [70]. This finding appears to contradict the impairment seen in Drp1-deficient models. The discrepancy likely reflects the distinction between acute pharmacological inhibition and chronic genetic deficiency. Mdivi-1 may produce a controlled reduction in fission that shifts the network toward a more fused, high-capacity respiratory configuration. This superficially resembles the fusion-biased state observed after prolonged training, but the two states are mechanistically distinct. Long-term training preserves Drp1 protein levels and retains fission capacity as a functional reserve, establishing a dynamic equilibrium that can respond rapidly to metabolic challenge or acute injury. By contrast, both Mdivi-1 treatment and chronic Drp1 deletion represent a loss of fission competence. The resulting static hyperfused network may temporarily enhance respiratory capacity but lacks the retained fission machinery required for quality control and adaptive remodeling. The metabolic output of Drp1 modulation therefore depends tightly on context, dose, and duration.

### 4.3. From Metabolic Adaptation to Exercise Performance

The metabolic rearrangements described above are closely linked to the muscle’s capacity to sustain contractile work and resist fatigue. Moore et al. provided direct evidence linking Drp1 function to exercise performance. Mice with muscle-specific Drp1 heterozygosity exhibited reduced maximal running speed and endurance capacity compared with wild-type littermates. Moreover, the improvement in time to fatigue during running following a training intervention was attenuated in these mice, suggesting that normal Drp1 expression may be required for the adaptive response to exercise [20]. Even a partial reduction in Drp1 levels, insufficient to cause overt muscle atrophy at the time of assessment, was enough to compromise both baseline performance and training-induced gains.

A putative mechanistic link between molecular defects and performance deficits can be inferred from the evidence reviewed above. Drp1 dysfunction impairs Sdhaf2-mediated complex II assembly, reducing electron transport chain capacity [8]. Simultaneously, the loss of appropriate fission may disrupt CPT1 regulation, potentially constraining fatty acid oxidation and increasing reliance on carbohydrate substrates [23]. The resulting impairment in substrate flux may contribute to lactate accumulation, lipid deposition, and reduced insulin sensitivity, all of which may promote premature fatigue [20]. When Drp1 deficiency is severe and sustained, the consequences become severe. In young C57BL/6J mice with AAV9-mediated *Dnm1l* knockdown, a 40–50% loss of muscle mass occurs over four months, accompanied by depressed ADP-stimulated mitochondrial respiration, impaired regenerative capacity, denervation, fibrosis, and elevated oxidative stress [17]. These long-term degenerative changes may reflect the endpoint of a process that begins with disordered mitochondrial dynamics and progresses through metabolic decline to structural deterioration.

The three-stage model describes how exercise-induced Drp1 regulation supports performance across timescales. Acute fission, by promoting fragmentation, may facilitate the metabolic shift toward fatty acid oxidation required during sustained contractile activity. Short-term coordinated upregulation of both fission and fusion machinery supports the turnover and expansion of the mitochondrial pool, enabling the muscle to meet the heightened energy demands of repeated training bouts. Long-term fusion-biased network remodeling, with increased cristae density and supercomplex assembly, may enhance oxidative phosphorylation efficiency, while the retention of fission capacity helps preserve the ability to respond rapidly to future metabolic challenges. Exercise performance is not solely a function of contractile protein isoforms or calcium handling. It also depends on the dynamic health of the mitochondrial network, with Drp1 linking mitochondrial morphology to metabolic capacity.

Taken together, these observations suggest that Drp1 sits at a critical junction where mitochondrial morphology, energy metabolism, and muscle function intersect. Under physiological conditions, exercise may tune Drp1 activity through the three-stage dynamic process and thereby contribute to improved performance. When the system is pushed away from this physiological range by chronic disease, however, Drp1 regulation breaks down in opposite directions. Metabolic disease drives excessive fission, while aging leads to insufficient fission. These two conditions represent opposite extremes on the fission activity spectrum and illustrate, in pathological form, the same Goldilocks principle that operates in physiological Drp1 regulation.

## 5. Drp1 in Skeletal Muscle Pathologies

### 5.1. The Role of Drp1 in the Development of Metabolic Diseases

In metabolic diseases such as diabetes and obesity, Drp1 dysregulation takes the form of chronic overactivation. These conditions, which have become major global public health burdens [71,72,73], are characterized by excessive mitochondrial fragmentation driven by persistently elevated Drp1 activity. Mitochondrial dysfunction has been widely recognized as a key pathological factor contributing to insulin resistance and impaired energy metabolism [74]. The regulation of mitochondrial fission, particularly the modulation of Drp1 activity, is considered a central mechanism linking nutritional excess to skeletal muscle metabolic dysfunction.

In diet-induced obesity, chronic overnutrition leads to persistent activation of Drp1 and excessive mitochondrial fragmentation [24]. In C57BL/6J mice fed a high-fat diet (HFD) for 12 weeks, the phosphorylation ratio of Drp1 at Ser616 to Ser637 rises significantly in skeletal muscle [25]. This shift is accompanied by impaired ADP-stimulated mitochondrial respiration and dysregulated autophagic signaling, reflected in reduced p62 protein abundance and an increased LC3B II/I ratio. Exercise can reverse this aberrant Drp1 activation. Ten weeks of high-intensity interval training (HIIT) reduced the HFD-induced increase in the Drp1 Ser616/Ser637 phosphorylation ratio by 35.7% and restored mitochondrial respiratory capacity and autophagic flux in mice [25]. In adults with obesity (BMI >30 kg/m^2^), 12 weeks of exercise training decreased Drp1 Ser616 phosphorylation in vastus lateralis muscle and improved substrate metabolism and insulin sensitivity [26]. These findings are consistent with the concept of a “Goldilocks zone” for Drp1 activity, in which either excessive or insufficient fission can compromise mitochondrial homeostasis. In obesity, Drp1 is chronically overactivated, thereby driving pathological mitochondrial fragmentation and mitochondrial dysfunction. Exercise training acts to suppress this excessive fission activity, thereby restoring network integrity and improving metabolic health.

Mechanistically, partial deletion of Drp1 in skeletal muscle of high-fat diet-fed mice has been shown to improve whole-body glucose tolerance and insulin sensitivity, while restoring mitochondrial morphology and reducing H_2_O_2_ production mediated by complex I and complex II [75]. In primary human myotubes derived from individuals with severe obesity and insulin resistance, *DNM1L* knockdown enhanced insulin signaling, stimulated glucose uptake, and reduced cellular ROS levels compared with control cells [75]. These findings support the notion that pathological Drp1 overactivation may contribute to mitochondrial dysfunction and insulin resistance in obesity. Moreover, interventions aimed at reducing excessive Drp1 activity, whether genetic or exercise-based, may ameliorate metabolic dysfunction.

Clinically, these observations have also been validated. In a randomized controlled trial, aerobic exercise training significantly reduced skeletal muscle Drp1 phosphorylation at Ser616 in patients with T2DM, independent of changes in PGC-1α and AMPK levels [76]. This reduction in Drp1 overactivation was accompanied by increased maximal NADH-linked oxidative phosphorylation capacity, improved succinate- and complex III-linked oxidative phosphorylation, and a marked shift toward elongated mitochondrial morphology with reduced sphericity. Exercise training reversed skeletal muscle Drp1 overactivation and improved respiratory capacity independently of mitochondrial biogenesis, indicating that improved mitochondrial dynamics is a major mechanism underlying the metabolic benefits of exercise in T2DM.

In summary, under metabolic disease conditions, Drp1 is chronically overactivated, leading to excessive mitochondrial fragmentation. Exercise training may suppress this pathological overactivation and help restore Drp1 activity toward a physiological range, thereby improving mitochondrial function and insulin sensitivity.

### 5.2. Drp1 Dysregulation in Aging

In contrast to the hyperactivated state observed in metabolic disease, aging skeletal muscle exhibits a fundamentally different pattern of Drp1 dysregulation [77]. With advancing age, skeletal muscle undergoes a progressive decline in muscle mass, strength, and function, a condition referred to as sarcopenia. Accumulating evidence indicates that impaired mitochondrial dynamics play a central role in this process [78].

In *Drosophila melanogaster*, Drp1 levels decline with age [28]. In C57BL/6 mice, Drp1 reduction occurs between 6 and 24 months of age, before overt muscle atrophy becomes apparent [29]. Notably, the expression of the mitochondrial fusion-related proteins OPA1, MFN1, and MFN2 is also decreased in aged mice [29], suggesting that both the fission and fusion arms of the mitochondrial dynamic machinery are suppressed during aging. Impaired fission capacity can lead to defective mitophagy [79], followed by the progressive accumulation of damaged and dysfunctional organelles. This pattern might appear to reflect a simple state of fission deficiency. However, in 24-month-old C57BL/6J mice, Dulac et al. demonstrated that both Drp1 knockdown and Drp1 overexpression impaired muscle and mitochondrial quality [27]. Aging does not merely reduce Drp1 expression but narrows the permissible window of Drp1 activity, rendering the system vulnerable to bidirectional perturbation.

It has been reported that Nrf2 mRNA expression is reduced in aged mice, whereas Nrf2-deficient C57BL/6J mice develop more severe sarcopenia, characterized by decreased mitochondrial biogenesis, as reflected by reduced PGC-1α, and impaired mitochondrial dynamics, as indicated by lower Drp1 and OPA1 levels [80,81]. The age-related decline in basal Drp1 expression therefore represents a drift toward the lower boundary of a window that has itself contracted. By restoring mitochondrial dynamics, OXPHOS function, and mitophagy, the regenerative capacity of satellite cells from aged C57BL/6J mice can be effectively improved, thereby alleviating muscle atrophy [82].

The organismal response to exercise in the context of aging also reflects this directional distinction. In sedentary older adults, Drp1 expression is reduced and muscle strength is decreased. In contrast, older athletes who maintain long-term physical activity exhibit preserved Drp1 expression and better muscle function [83]. At the gene-expression level, changes in genes encoding fusion- and fission-related proteins in aged skeletal muscle occur in parallel after long-term physical activity or endurance exercise training. For example, compared with age-matched inactive women, lifelong physically active older women show higher *MFN2* and *DNM1L* mRNA levels in skeletal muscle [83]. At the protein level, resistance training in older adults increases Drp1 expression, along with the expression of the fusion proteins MFN1, MFN2, and OPA1. These changes are consistent with concomitant increases in OXPHOS-related proteins [22]. In addition, in aged rats, a 12-week exercise intervention increases PGC-1α, MFN2, Drp1, and PINK1 levels, thereby improving mitochondrial function and suppressing the progression of sarcopenia [84]. These exercise-induced increases in Drp1 expression are therefore not merely compensatory but restorative, moving Drp1 activity back from the contracted lower boundary toward the center of a narrowed permissible window.

The molecular basis underlying exercise-induced restoration of Drp1 function during aging has been partially elucidated. ROS generated by moderate-intensity exercise can activate the Nrf2 pathway, and Nrf2, in turn, regulates Drp1 stability and activity by promoting Drp1 deubiquitination [30]. This regulatory axis facilitates moderate mitochondrial fission, thereby maintaining mitochondrial health and slowing the progression of age-related sarcopenia. Unlike in metabolic disease where exercise suppresses excessive Drp1 activity, in aging, exercise restores deficient Drp1 function. Importantly, the requirement for precision is heightened in aged muscle because the permissible window has narrowed. Exercise must restore Drp1 activity without overshooting into the hyperactivation range, a challenge that demands precisely tuned Drp1 regulation.

### 5.3. Convergent Pathology from Divergent Drp1 Dysregulation

The patterns of Drp1 dysregulation in metabolic disease and aging are mirror images of each other. In obesity and type 2 diabetes, chronic nutrient excess drives Drp1 hyperactivation, producing excessive mitochondrial fragmentation, impaired respiratory capacity, and insulin resistance. In aging, Drp1 expression and activity are deficient, resulting in mitochondrial swelling, defective mitophagy, and accumulation of dysfunctional organelles. Despite these opposite molecular signatures, the functional endpoints are strikingly similar. Both conditions lead to mitochondrial dysfunction, muscle atrophy, and compromised exercise capacity. This convergence suggests that the absolute level of Drp1 activity is less important than its position relative to a physiological set point. The question arising from this observation is how exercise can correct deviations in both directions. The mechanistic basis for this bidirectional capacity lies in the ROS-Drp1 signaling axis.

## 6. The ROS-Drp1 Axis as a Bidirectional Actuator of Exercise Adaptation

### 6.1. ROS Hormesis and the Drp1 Goldilocks Zone

Reactive oxygen species are generated in skeletal muscle during contraction from multiple sources, including mitochondria, NADPH oxidases, and xanthine oxidase [85]. Rather than being exclusively deleterious, ROS at moderate concentrations serve as signaling molecules that activate adaptive programs, whereas excessive ROS overwhelm antioxidant defenses and cause oxidative damage [86,87]. This biphasic dose–response relationship, termed mitohormesis, is central to understanding how exercise regulates Drp1.

The relationship between ROS and Drp1 is bidirectional. Exogenous H_2_O_2_ triggers mitochondrial depolarization and fragmentation in cultured myoblasts within hours, and co-treatment with the Drp1 inhibitor Mdivi-1 reduces this fragmentation [88], indicating that ROS act directly on the fission machinery. Conversely, mitochondrial fission itself can promote further ROS production [89], creating a potential feedback loop. The direction and outcome of this loop depend critically on the magnitude and duration of the ROS signal.

When ROS production is moderate and transient, as occurs during an acute exercise bout, Drp1-mediated fission supports quality control by enabling the segregation and clearance of damaged mitochondrial segments through mitophagy [19,90]. This process is complemented by the activation of adaptive stress responses, including the mitochondrial unfolded protein response and the endoplasmic reticulum unfolded protein response, which together restore proteostasis and maintain mitochondrial function [91,92]. As ROS levels decline during recovery, the mitochondrial network reassembles through fusion, completing a cycle of selective degradation and network reassembly [57].

**Figure 4:**
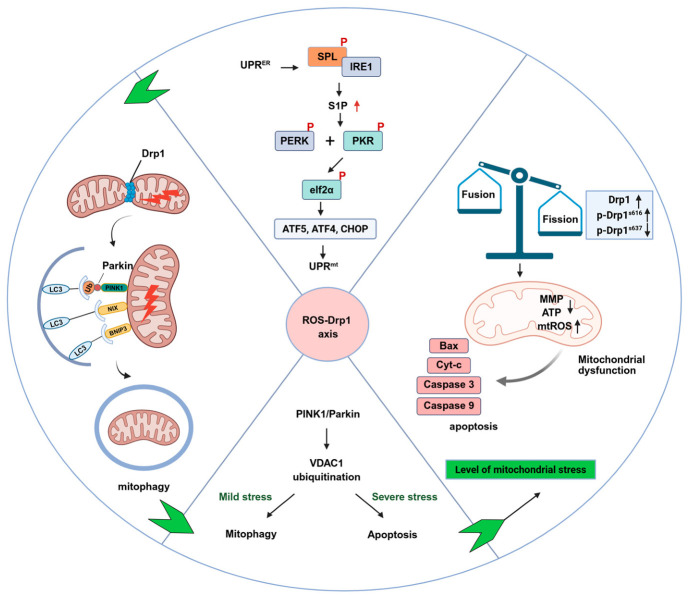
Role of the ROS-Drp1 axis in the mitochondrial stress response. Under mild stress conditions, moderate levels of ROS regulate Drp1-mediated mitochondrial fission and activate adaptive stress responses, including the endoplasmic reticulum unfolded protein response (ER-UPR) and the mitochondrial unfolded protein response (mtUPR), thereby maintaining mitochondrial function. When mitochondrial damage exceeds the compensatory capacity of these responses, mitophagy is activated to selectively remove damaged or depolarized mitochondria. As stress intensity further increases, excessive ROS drive sustained Drp1 activation and excessive mitochondrial fragmentation, accompanied by mitochondrial membrane potential loss, reduced ATP production, and increased mitochondrial ROS generation. When mitochondrial damage surpasses the protective capacity of mitophagy and UPR signaling, the cellular response shifts from adaptive quality control to mitochondria-dependent apoptosis. The intensity and duration of ROS-Drp1 signaling therefore determine whether mitochondrial fission contributes to adaptive quality control or promotes maladaptive mitochondrial dysfunction and apoptosis. Arrows indicate the direction of signaling or pathological processes, as well as increases or decreases in the indicated molecules or mitochondrial functions; the red letter “P” denotes phosphorylation. Created with BioRender.com under a publication license.

When ROS production is chronically elevated, however, as in metabolic disease, the balance shifts toward pathology. Persistent oxidative stress drives sustained Drp1 activation, leading to excessive fragmentation, loss of inner membrane integrity, impaired oxidative phosphorylation, and, if the damage exceeds the protective capacity of mitophagy and UPR signaling, activation of mitochondria-dependent apoptosis [93,94]. The ROS-Drp1 axis therefore operates as a cellular stress integrator. Moderate ROS signals promote adaptive fission and quality control, whereas excessive or insufficient ROS signaling leads to maladaptive fragmentation or impaired clearance, respectively (Figure 4).

This biphasic relationship establishes a functional Goldilocks zone for Drp1 activity. Within this zone, Drp1-mediated fission supports mitochondrial quality control and metabolic adaptation without triggering pathological fragmentation. A key mediator that couples physiological ROS production to Drp1 regulation is the transcription factor Nrf2. Exercise-induced ROS activate Nrf2, which in turn promotes Drp1 deubiquitination and stabilizes the protein, thereby maintaining fission competence [30]. In aged skeletal muscle, where basal Nrf2 signaling declines and Drp1 expression falls, this pathway becomes critical for preserving mitochondrial health. Nrf2 knockout mice develop more severe sarcopenia with reduced Drp1 levels, and the beneficial effects of exercise on muscle function are markedly attenuated in these animals [30,81]. The ROS-Nrf2-Drp1 axis thus represents a signaling pathway through which exercise-induced redox signaling is transduced into appropriate mitochondrial fission. Beyond this acute regulation, exercise-induced ROS also drives long-term adaptive programming through epigenetic mechanisms. Our group has recently shown that endurance exercise activates the ROS/AMPK pathway to remodel histone methylation at the promoters of PGC-1α and myosin heavy chain isoforms in rat skeletal muscle, thereby coupling mitochondrial biogenesis with a shift toward an oxidative muscle fiber phenotype [95]. These findings establish exercise-induced ROS as a signal that coordinates both the rapid tuning of mitochondrial dynamics and the sustained reprogramming of metabolic gene expression in skeletal muscle.

### 6.2. Context-Dependent Bidirectional Regulation by Exercise

A striking feature of exercise as a physiological intervention is its ability to regulate Drp1 activity in opposite directions depending on the basal pathological state. This bidirectional capacity distinguishes exercise from pharmacological inhibitors, which typically produce unidirectional suppression regardless of context.

In obesity and type 2 diabetes, Drp1 is chronically hyperactivated, a state characterized by elevated Ser616 phosphorylation, excessive mitochondrial fragmentation, and impaired respiratory capacity. Exercise training suppresses this pathological activation, an effect documented consistently across rodent models and human trials [25,26,76]. Mechanistically, this suppression is mediated in part by AMPK signaling. In a rat model of type 2 diabetes induced by a high-fat diet and streptozotocin, exercise-induced AMPK activation reduces expression of fission-related proteins including Drp1, Fis1, and MFF, while increasing the fusion protein MFN2, thereby alleviating diabetes-induced insulin resistance [72]. mTORC1 also contributes to Drp1 regulation. Morita et al. demonstrated that mTORC1 stimulates translation of the fission protein MTFP1 through 4E-BP, and that MTFP1 promotes Drp1 recruitment to mitochondria and its phosphorylation at Ser616 [96]. Exercise-induced mTORC1 inhibition may therefore complement AMPK signaling in restraining excessive fission in metabolic disease.

Aging skeletal muscle shows the opposite pattern. Drp1 expression and activity are deficient, a decline accompanied by reduced expression of OPA1, MFN1, and MFN2 [28,29]. Impaired fission capacity leads to defective mitophagy and progressive accumulation of damaged organelles. In this context, exercise restores Drp1 function. Lifelong physical activity preserves Drp1 and MFN2 expression in older adults, and resistance training elevates Drp1 alongside fusion proteins [22,83,84]. The molecular basis for this restoration involves the ROS-Nrf2 axis. Moderate exercise-induced ROS production activates Nrf2, which promotes Drp1 deubiquitination and enhances protein stability, thereby supporting appropriate fission activity and mitochondrial quality control [30].

The distinct reliance on AMPK in metabolic disease and Nrf2 in aging is not arbitrary. It reflects how the same exercise-generated ROS signal is interpreted in opposite metabolic contexts. In obesity and type 2 diabetes, chronic nutrient excess maintains Drp1 in a hyperactivated state driven by persistent oxidative stress. Exercise-induced AMPK activation directly opposes this hyperactivation by phosphorylating Drp1 at Ser637 and by reducing expression of fission-related proteins. Nrf2 is also activated by exercise in this context, but its effect on Drp1 is overridden by the dominant AMPK-driven suppression of fission because the primary defect is excess. In aging, the situation is reversed. Basal Nrf2 signaling declines with age, and Drp1 expression falls because its protein stability depends on Nrf2-mediated deubiquitination. Exercise-induced ROS activate Nrf2 and restore Drp1 protein levels. AMPK is likewise activated, but its inhibitory phosphorylation of Drp1 is offset by the Nrf2-driven recovery of Drp1 abundance. The quantitative balance between these opposing effects remains to be determined, but the net restorative outcome observed in aged muscle following exercise training [22,83,84] indicates that the Nrf2-driven recovery of Drp1 abundance predominates under these conditions. The two pathways are therefore differentially engaged depending on the prevailing pathological state, which determines the net effect of exercise-induced ROS signaling on Drp1 activity.

**Figure 5:**
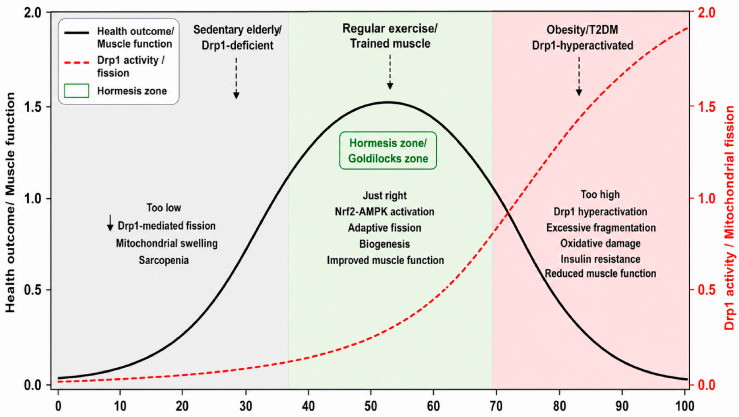
Role of the ROS-Drp1 axis in the mitochondrial stress response: a biphasic dose–response relationship. Low ROS levels (e.g., sedentary aging) result in insufficient Drp1-mediated fission, leading to mitochondrial swelling and sarcopenia. Moderate ROS levels induced by regular exercise activate Nrf2-AMPK signaling, adjusting Drp1 activity into the physiological “Goldilocks zone”, which supports adaptive fission, mitophagy, and mitochondrial biogenesis. Excessive ROS (e.g., obesity, type 2 diabetes) drive pathological Drp1 hyperactivation, causing excessive fragmentation, oxidative damage, and insulin resistance. The shaded green area represents the hormetic zone where exercise-induced ROS promote beneficial adaptations. The left, middle, and right vertical dashed arrows indicate “Sedentary elderly/Drp1-deficient”, “Regular exercise/Trained muscle”, and “Obesity/T2DM/Drp1-hyperactivated”, respectively; the solid downward arrow denotes decreased Drp1-mediated mitochondrial fission.

The capacity of exercise to suppress excessive Drp1 activity in metabolic disease while restoring deficient Drp1 activity in aging reveals context-dependent regulation. Rather than acting as a simple activator or inhibitor of Drp1, exercise acts as a homeostatic regulator that responds to the prevailing state of the mitochondrial network and adjusts fission activity toward the physiological Goldilocks zone. This bidirectional regulation, mediated by the ROS-Nrf2 and ROS-AMPK signaling axes, represents a therapeutic capability that current pharmacological approaches cannot replicate (Figure 5).

## 7. Conclusions and Perspectives

The evidence reviewed here identifies Drp1 as a bidirectional Goldilocks integrator of exercise-induced mitochondrial adaptations in skeletal muscle. Three principles support this view. First, Drp1 activity must stay within a narrow physiological range. Both deficiency and overabundance produce muscle atrophy and dysfunction. Second, Drp1 is regulated by multiple post-translational modifications. Phosphorylation at serine 616 and serine 637 provides canonical activating and inhibitory signals, while methylation, S-nitrosylation, acetylation, and ubiquitination broaden the regulatory landscape. Distinct receptors may further specify context-dependent fission programs.

Exercise training regulates Drp1 across three temporal stages. Acute exercise triggers Drp1 serine 616 phosphorylation and transient mitochondrial fragmentation, initiating quality control. Short-term training coordinately upregulates fission and fusion proteins, enabling expansion and renewal of the mitochondrial pool. Long-term training produces a hyperfused, cristae-dense network optimized for oxidative efficiency while retaining fission capacity. How Drp1 and Mfn2 coordinate their activities during these three stages remains poorly understood. This process is supported by the ROS-Nrf2-Drp1 and ROS-AMPK-Drp1 axes. Moderate exercise-induced ROS activate Nrf2, which promotes Drp1 deubiquitination and stabilizes the protein, while AMPK modulates the Ser616/Ser637 phosphorylation balance and adjusts fission and fusion protein expression. Direct redox modifications of Drp1 cysteine residues, such as S-nitrosylation at cysteine 644, may provide an additional control, although this remains largely unexplored in skeletal muscle.

**Figure 6:**
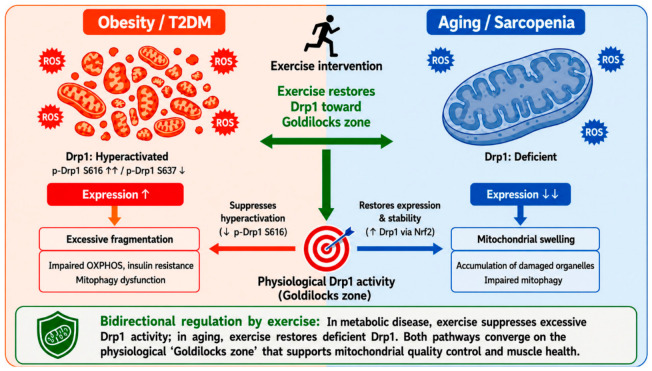
Bidirectional regulation of Drp1 by exercise in metabolic disease and aging. (**Left**) panel: In obesity and type 2 diabetes, chronic overnutrition drives Drp1 hyperactivation (elevated Ser616 phosphorylation and protein expression), leading to excessive mitochondrial fragmentation, impaired oxidative phosphorylation, insulin resistance, and mitophagy dysfunction. (**Right**) panel: In aging skeletal muscle, Drp1 expression and activity are deficient, resulting in mitochondrial swelling, accumulation of damaged organelles, and impaired clearance. Exercise training (**center**) exerts bidirectional effects: it suppresses Drp1 hyperactivation in metabolic disease (e.g., reducing p-Drp1 Ser616) and restores Drp1 expression and stability in aging (e.g., via Nrf2-mediated deubiquitination). Both actions move Drp1 activity toward the physiological “Goldilocks zone”, where mitochondrial fission supports quality control without causing pathological fragmentation or swelling. This framework highlights exercise as a context-dependent integrator of Drp1 function. Green arrows indicate exercise-mediated bidirectional regulation toward the physiological Drp1 “Goldilocks zone”; red/orange arrows indicate suppression of Drp1 hyperactivation in obesity/T2DM; blue arrows indicate restoration of deficient Drp1 expression in aging/sarcopenia.

Exercise regulates Drp1 with a capacity for bidirectional control. In metabolic disease, exercise suppresses Drp1 hyperactivation and restores respiratory capacity. In aging, exercise restores Drp1 expression and fission activity, in part through Nrf2-mediated protein stabilization. This context-dependent regulation distinguishes exercise from pharmacological inhibitors, which produce unidirectional inhibition regardless of the underlying state. The observation that both Drp1 knockdown and overexpression are detrimental in aged muscle [27] further shows that aged muscle tolerates deviations from the physiological set point poorly in either direction (Figure 6).

Several outstanding questions require future research. The temporal dynamics of the Drp1 PTM landscape across exercise training stages have not been systematically mapped. Phosphoproteomic and ubiquitinomic approaches applied to skeletal muscle biopsies across defined training intervals could reveal the sequence and interdependence of modifications that establish the three-stage process. The receptor selectivity hypothesis, supported by circumstantial evidence from cardiac and aging muscle, has not been tested directly through skeletal muscle-specific conditional knockout models targeting individual receptors. Translational development of the ROS-Drp1 Goldilocks concept faces challenges of tissue specificity and therapeutic window. Nrf2 activators such as sulforaphane have shown promise in preclinical models, yet not all metabolic modulators are exercise mimetics. Metformin suppresses the mitochondrial and transcriptional response to training [97]. Defining the exercise intensity and duration that optimizes Drp1 activity in each patient population, from sarcopenic elderly to individuals with type 2 diabetes, may require biomarkers such as Drp1 phosphorylation status in peripheral blood mononuclear cells and could lead toward personalized exercise prescriptions.

## Data Availability

No new data were created or analyzed in this study. Data sharing is not applicable to this article.

## Abbreviations

The following abbreviations are used in this manuscript:

Drp1	Dynamin-related protein 1
ROS	Reactive oxygen species
Nrf2	Nuclear factor erythroid 2-related factor 2
AMPK	AMP-activated protein kinase
PGC-1α	Peroxisome proliferator-activated receptor gamma coactivator 1-alpha
CPT1	Carnitine palmitoyltransferase 1
Sdhaf2	Succinate dehydrogenase assembly factor 2
MFN1/2	Mitofusin 1/2
OPA1	Optic atrophy 1
mTORC1/2	Mechanistic target of rapamycin complex 1/2
GDF-15	Growth differentiation factor 15
MiD49	Mitochondrial dynamics proteins 49
MiD51	Mitochondrial dynamics proteins 51
MFF	Mitochondrial fission factor
Mfn2	Mitofusin 2
MTFP1	Mitochondrial fission process 1
Fis1	Mitochondrial fission 1 protein
PTMs	post-translational modifications
PKA	Protein kinase A
CARM1	Coactivator-associated arginine methyltransferase 1
PKCδ	Protein kinase C delta
PINK1	PTEN-induced kinase 1
Parkin	Parkin RBR E3 ubiquitin protein ligase
Mdivi-1	Mitochondrial division inhibitor 1
HIIT	High-intensity interval training
ER-UPR	Endoplasmic reticulum unfolded protein response
mtUPR	Mitochondrial unfolded protein response

## References

[1] Axelrod C.L., Fealy C.E., Mulya A., Kirwan J.P. (2019). Exercise training remodels human skeletal muscle mitochondrial fission and fusion machinery towards a pro-elongation phenotype. Acta Physiol..

[2] Nielsen J., Gejl K.D., Hey-Mogensen M., Holmberg H.C., Suetta C., Krustrup P., Elemans C.P.H., Ortenblad N. (2017). Plasticity in mitochondrial cristae density allows metabolic capacity modulation in human skeletal muscle. J. Physiol..

[3] Greggio C., Jha P., Kulkarni S.S., Lagarrigue S., Broskey N.T., Boutant M., Wang X., Conde Alonso S., Ofori E., Auwerx J. (2017). Enhanced Respiratory Chain Supercomplex Formation in Response to Exercise in Human Skeletal Muscle. Cell Metab..

[4] Gusdon A.M., Callio J., Distefano G., O’Doherty R.M., Goodpaster B.H., Coen P.M., Chu C.T. (2017). Exercise increases mitochondrial complex I activity and DRP1 expression in the brains of aged mice. Exp. Gerontol..

[5] Jin J.Y., Wei X.X., Zhi X.L., Wang X.H., Meng D. (2021). Drp1-dependent mitochondrial fission in cardiovascular disease. Acta Pharmacol. Sin..

[6] Adebayo M., Singh S., Singh A.P., Dasgupta S. (2021). Mitochondrial fusion and fission: The fine-tune balance for cellular homeostasis. FASEB J..

[7] Rambold A.S., Pearce E.L. (2018). Mitochondrial Dynamics at the Interface of Immune Cell Metabolism and Function. Trends Immunol..

[8] Zhou Z., Ma A., Moore T.M., Wolf D.M., Yang N., Tran P., Segawa M., Strumwasser A.R., Ren W., Fu K. (2024). Drp1 controls complex II assembly and skeletal muscle metabolism by Sdhaf2 action on mitochondria. Sci. Adv..

[9] Nagai Y., Matoba K., Nishimura R. (2026). ROCK signaling at the crossroads of redox stress, mitochondrial dynamics, and metabolic disease. Redox Biol..

[10] Nivedya C., Venkhatesh P., Rodriguez B.I., Le H., Afolabi J., Marshall A., Neikirk K., Masenga S.K., Aftab M., Kazma L.J. (2025). Roles of DRP1 and the fission protein interactome as regulators of cellular stability and sarcopenia in skeletal muscle aging. Aging Adv..

[11] Bo T., Yamamori T., Suzuki M., Sakai Y., Yamamoto K., Inanami O. (2018). Calmodulin-dependent protein kinase II (CaMKII) mediates radiation-induced mitochondrial fission by regulating the phosphorylation of dynamin-related protein 1 (Drp1) at serine 616. Biochem. Biophys. Res. Commun..

[12] Adhikary A., Mukherjee A., Banerjee R., Nagotu S. (2023). DRP1: At the Crossroads of Dysregulated Mitochondrial Dynamics and Altered Cell Signaling in Cancer Cells. ACS Omega.

[13] Strack S., Cribbs J.T. (2012). Allosteric modulation of Drp1 mechanoenzyme assembly and mitochondrial fission by the variable domain. J. Biol. Chem..

[14] Cho Y., Kim Y.K. (2024). ROS-mediated cytoplasmic localization of CARM1 induces mitochondrial fission through DRP1 methylation. Redox Biol..

[15] Kraus F., Roy K., Pucadyil T.J., Ryan M.T. (2021). Function and regulation of the divisome for mitochondrial fission. Nature.

[16] Favaro G., Romanello V., Varanita T., Andrea Desbats M., Morbidoni V., Tezze C., Albiero M., Canato M., Gherardi G., De Stefani D. (2019). DRP1-mediated mitochondrial shape controls calcium homeostasis and muscle mass. Nat. Commun..

[17] Dulac M., Leduc-Gaudet J.P., Reynaud O., Ayoub M.B., Guerin A., Finkelchtein M., Hussain S.N., Gouspillou G. (2020). Drp1 knockdown induces severe muscle atrophy and remodelling, mitochondrial dysfunction, autophagy impairment and denervation. J. Physiol..

[18] Touvier T., De Palma C., Rigamonti E., Scagliola A., Incerti E., Mazelin L., Thomas J.L., D’Antonio M., Politi L., Schaeffer L. (2015). Muscle-specific Drp1 overexpression impairs skeletal muscle growth via translational attenuation. Cell Death Dis..

[19] Laker R.C., Drake J.C., Wilson R.J., Lira V.A., Lewellen B.M., Ryall K.A., Fisher C.C., Zhang M., Saucerman J.J., Goodyear L.J. (2017). Ampk phosphorylation of Ulk1 is required for targeting of mitochondria to lysosomes in exercise-induced mitophagy. Nat. Commun..

[20] Moore T.M., Zhou Z., Cohn W., Norheim F., Lin A.J., Kalajian N., Strumwasser A.R., Cory K., Whitney K., Ho T. (2019). The impact of exercise on mitochondrial dynamics and the role of Drp1 in exercise performance and training adaptations in skeletal muscle. Mol. Metab..

[21] Ju J.S., Jeon S.I., Park J.Y., Lee J.Y., Lee S.C., Cho K.J., Jeong J.M. (2016). Autophagy plays a role in skeletal muscle mitochondrial biogenesis in an endurance exercise-trained condition. J. Physiol. Sci..

[22] Mesquita P.H.C., Lamb D.A., Parry H.A., Moore J.H., Smith M.A., Vann C.G., Osburn S.C., Fox C.D., Ruple B.A., Huggins K.W. (2020). Acute and chronic effects of resistance training on skeletal muscle markers of mitochondrial remodeling in older adults. Physiol. Rep..

[23] Ngo J., Choi D.W., Stanley I.A., Stiles L., Molina A.J.A., Chen P.H., Lako A., Sung I.C.H., Goswami R., Kim M.Y. (2023). Mitochondrial morphology controls fatty acid utilization by changing CPT1 sensitivity to malonyl-CoA. EMBO J..

[24] Gasier H.G., Dohl J., Suliman H.B., Piantadosi C.A., Yu T. (2020). Skeletal muscle mitochondrial fragmentation and impaired bioenergetics from nutrient overload are prevented by carbon monoxide. Am. J. Physiol. Cell Physiol..

[25] Tincknell J.B., Kugler B.A., Spicuzza H., Berger N., Yan H., You T., Zou K. (2024). High-intensity interval training attenuates impairment in regulatory protein machinery of mitochondrial quality control in skeletal muscle of diet-induced obese mice. Appl. Physiol. Nutr. Metab..

[26] Fealy C.E., Mulya A., Lai N., Kirwan J.P. (2014). Exercise training decreases activation of the mitochondrial fission protein dynamin-related protein-1 in insulin-resistant human skeletal muscle. J. Appl. Physiol..

[27] Dulac M., Leduc-Gaudet J.P., Cefis M., Ayoub M.B., Reynaud O., Shams A., Moamer A., Nery Ferreira M.F., Hussain S.N., Gouspillou G. (2021). Regulation of muscle and mitochondrial health by the mitochondrial fission protein Drp1 in aged mice. J. Physiol..

[28] Rana A., Oliveira M.P., Khamoui A.V., Aparicio R., Rera M., Rossiter H.B., Walker D.W. (2017). Promoting Drp1-mediated mitochondrial fission in midlife prolongs healthy lifespan of Drosophila melanogaster. Nat. Commun..

[29] Del Campo A., Contreras-Hernandez I., Castro-Sepulveda M., Campos C.A., Figueroa R., Tevy M.F., Eisner V., Casas M., Jaimovich E. (2018). Muscle function decline and mitochondria changes in middle age precede sarcopenia in mice. Aging.

[30] Yan X., Shen Z., Yu D., Zhao C., Zou H., Ma B., Dong W., Chen W., Huang D., Yu Z. (2022). Nrf2 contributes to the benefits of exercise interventions on age-related skeletal muscle disorder via regulating Drp1 stability and mitochondrial fission. Free Radic. Biol. Med..

[31] Mishra P., Varuzhanyan G., Pham A.H., Chan D.C. (2015). Mitochondrial Dynamics is a Distinguishing Feature of Skeletal Muscle Fiber Types and Regulates Organellar Compartmentalization. Cell Metab..

[32] Tezze C., Romanello V., Desbats M.A., Fadini G.P., Albiero M., Favaro G., Ciciliot S., Soriano M.E., Morbidoni V., Cerqua C. (2017). Age-Associated Loss of OPA1 in Muscle Impacts Muscle Mass, Metabolic Homeostasis, Systemic Inflammation, and Epithelial Senescence. Cell Metab..

[33] Romanello V., Scalabrin M., Albiero M., Blaauw B., Scorrano L., Sandri M. (2019). Inhibition of the Fission Machinery Mitigates OPA1 Impairment in Adult Skeletal Muscles. Cells.

[34] Yasuda T., Ishihara T., Ichimura A., Ishihara N. (2023). Mitochondrial dynamics define muscle fiber type by modulating cellular metabolic pathways. Cell Rep..

[35] Burte F., Carelli V., Chinnery P.F., Yu-Wai-Man P. (2015). Disturbed mitochondrial dynamics and neurodegenerative disorders. Nat. Rev. Neurol..

[36] Jenner A., Pena-Blanco A., Salvador-Gallego R., Ugarte-Uribe B., Zollo C., Ganief T., Bierlmeier J., Mund M., Lee J.E., Ries J. (2022). DRP1 interacts directly with BAX to induce its activation and apoptosis. EMBO J..

[37] Chernyavskij D.A., Lyamzaev K.G., Pletjushkina O.Y., Chen F., Karpukhina A., Vassetzky Y.S., Chernyak B.V., Popova E.N. (2024). Mitochondrial fragmentation in early differentiation of human MB135 myoblasts: Role of mitochondrial ROS production in the absence of depolarization. Life Sci..

[38] Taguchi N., Ishihara N., Jofuku A., Oka T., Mihara K. (2007). Mitotic phosphorylation of dynamin-related GTPase Drp1 participates in mitochondrial fission. J. Biol. Chem..

[39] Chang C.R., Blackstone C. (2007). Cyclic AMP-dependent protein kinase phosphorylation of Drp1 regulates its GTPase activity and mitochondrial morphology. J. Biol. Chem..

[40] Diaz-Castro F., Tunon-Suarez M., Rivera P., Botella J., Cancino J., Figueroa A.M., Gutierrez J., Cantin C., Deldicque L., Zbinden-Foncea H. (2024). A single bout of resistance exercise triggers mitophagy, potentially involving the ejection of mitochondria in human skeletal muscle. Acta Physiol..

[41] Cai T., Li Y., Zhang Y., Li C., Li S., Zhang Q. (2026). The role of exercise-mediated mitochondrial quality control remodeling in aging. Front. Cell Dev. Biol..

[42] Cho D.H., Nakamura T., Fang J., Cieplak P., Godzik A., Gu Z., Lipton S.A. (2009). S-nitrosylation of Drp1 mediates beta-amyloid-related mitochondrial fission and neuronal injury. Science.

[43] Nakamura T., Lipton S.A. (2011). Redox modulation by S-nitrosylation contributes to protein misfolding, mitochondrial dynamics, and neuronal synaptic damage in neurodegenerative diseases. Cell Death Differ..

[44] Qi X., Disatnik M.H., Shen N., Sobel R.A., Mochly-Rosen D. (2011). Aberrant mitochondrial fission in neurons induced by protein kinase Cdelta under oxidative stress conditions in vivo. Mol. Biol. Cell.

[45] Abuarab N., Munsey T.S., Jiang L.H., Li J., Sivaprasadarao A. (2017). High glucose-induced ROS activates TRPM2 to trigger lysosomal membrane permeabilization and Zn(2+)-mediated mitochondrial fission. Sci. Signal.

[46] Xi Y., Tao K., Wen X., Feng D., Mai Z., Ding H., Mao H., Wang M., Yang Q., Xiang J. (2025). SIRT3-Mediated Deacetylation of DRP1(K711) Prevents Mitochondrial Dysfunction in Parkinson’s Disease. Adv. Sci..

[47] Lutz A.K., Exner N., Fett M.E., Schlehe J.S., Kloos K., Lammermann K., Brunner B., Kurz-Drexler A., Vogel F., Reichert A.S. (2009). Loss of parkin or PINK1 function increases Drp1-dependent mitochondrial fragmentation. J. Biol. Chem..

[48] Gao T., Shi R., Liu Z., De D., Li R., Chen Y., Pei J., Ding M. (2023). Ischemia/reperfusion-induced MiD51 upregulation recruits Drp1 to mitochondria and contributes to myocardial injury. Biochem. Biophys. Res. Commun..

[49] Loson O.C., Song Z., Chen H., Chan D.C. (2013). Fis1, Mff, MiD49, and MiD51 mediate Drp1 recruitment in mitochondrial fission. Mol. Biol. Cell.

[50] Voos K.M., Tzeng J., Patel P., Rubinsky S., Choi H.E., Pharr T., Sookram S., Baur J.A., Soderblom E.J., Lorenzo D.N. (2025). Ankyrin-B modulates mitochondrial fission in skeletal muscle and is required for optimal endurance exercise capacity. Nat. Commun..

[51] Wyckelsma V.L., Levinger I., McKenna M.J., Formosa L.E., Ryan M.T., Petersen A.C., Anderson M.J., Murphy R.M. (2017). Preservation of skeletal muscle mitochondrial content in older adults: Relationship between mitochondria, fibre type and high-intensity exercise training. J. Physiol..

[52] Ihenacho U.K., Toro R., Mansour R.H., Hill R.B. (2023). A conserved, noncanonical insert in FIS1 mediates TBC1D15 and DRP1 recruitment for mitochondrial fission. J. Biol. Chem..

[53] Ding H., Jiang N., Liu H., Liu X., Liu D., Zhao F., Wen L., Liu S., Ji L.L., Zhang Y. (2010). Response of mitochondrial fusion and fission protein gene expression to exercise in rat skeletal muscle. Biochim. Biophys. Acta.

[54] Caino M.C., Seo J.H., Aguinaldo A., Wait E., Bryant K.G., Kossenkov A.V., Hayden J.E., Vaira V., Morotti A., Ferrero S. (2016). A neuronal network of mitochondrial dynamics regulates metastasis. Nat. Commun..

[55] Hirokawa N., Takemura R. (2005). Molecular motors and mechanisms of directional transport in neurons. Nat. Rev. Neurosci..

[56] Schwalm C., Deldicque L., Francaux M. (2017). Lack of Activation of Mitophagy during Endurance Exercise in Human. Med. Sci. Sports Exerc..

[57] Khalilimeybodi A., Qiao L., Leung A., McCulloch A.D., Schenk S., Rangamani P. (2025). Systems modeling of mitochondrial dynamics in different exercise regimes. bioRxiv.

[58] Iqbal S., Ostojic O., Singh K., Joseph A.M., Hood D.A. (2013). Expression of mitochondrial fission and fusion regulatory proteins in skeletal muscle during chronic use and disuse. Muscle Nerve.

[59] Hernandez-Alvarez M.I., Thabit H., Burns N., Shah S., Brema I., Hatunic M., Finucane F., Liesa M., Chiellini C., Naon D. (2010). Subjects with early-onset type 2 diabetes show defective activation of the skeletal muscle PGC-1alpha/Mitofusin-2 regulatory pathway in response to physical activity. Diabetes Care.

[60] Drake J.C., Wilson R.J., Yan Z. (2016). Molecular mechanisms for mitochondrial adaptation to exercise training in skeletal muscle. FASEB J..

[61] Montero D., Cathomen A., Jacobs R.A., Fluck D., de Leur J., Keiser S., Bonne T., Kirk N., Lundby A.K., Lundby C. (2018). Haematological rather than skeletal muscle adaptations contribute to the increase in peak oxygen uptake induced by moderate endurance training. J. Physiol..

[62] Egan B., Zierath J.R. (2013). Exercise metabolism and the molecular regulation of skeletal muscle adaptation. Cell Metab..

[63] Lundby C., Jacobs R.A. (2016). Adaptations of skeletal muscle mitochondria to exercise training. Exp. Physiol..

[64] Philp A.M., Saner N.J., Lazarou M., Ganley I.G., Philp A. (2021). The influence of aerobic exercise on mitochondrial quality control in skeletal muscle. J. Physiol..

[65] Huertas J.R., Al Fazazi S., Hidalgo-Gutierrez A., Lopez L.C., Casuso R.A. (2017). Antioxidant effect of exercise: Exploring the role of the mitochondrial complex I superassembly. Redox Biol..

[66] Koves T.R., Noland R.C., Bates A.L., Henes S.T., Muoio D.M., Cortright R.N. (2005). Subsarcolemmal and intermyofibrillar mitochondria play distinct roles in regulating skeletal muscle fatty acid metabolism. Am. J. Physiol. Cell Physiol..

[67] Chilibeck P.D., Syrotuik D.G., Bell G.J. (2002). The effect of concurrent endurance and strength training on quantitative estimates of subsarcolemmal and intermyofibrillar mitochondria. Int. J. Sports Med..

[68] Chen C.C.W., Erlich A.T., Hood D.A. (2018). Role of Parkin and endurance training on mitochondrial turnover in skeletal muscle. Skelet. Muscle.

[69] Ribas V., Drew B.G., Zhou Z., Phun J., Kalajian N.Y., Soleymani T., Daraei P., Widjaja K., Wanagat J., de Aguiar Vallim T.Q. (2016). Skeletal muscle action of estrogen receptor alpha is critical for the maintenance of mitochondrial function and metabolic homeostasis in females. Sci. Transl. Med..

[70] Rexius-Hall M.L., Khalil N.N., Andres A.M., McCain M.L. (2020). Mitochondrial division inhibitor 1 (mdivi-1) increases oxidative capacity and contractile stress generated by engineered skeletal muscle. FASEB J..

[71] Wu X., Xu M., Geng M., Chen S., Little P.J., Xu S., Weng J. (2023). Targeting protein modifications in metabolic diseases: Molecular mechanisms and targeted therapies. Signal Transduct. Target. Ther..

[72] Lin J., Zhang X., Sun Y., Xu H., Li N., Wang Y., Tian X., Zhao C., Wang B., Zhu B. (2024). Author Correction: Exercise ameliorates muscular excessive mitochondrial fission, insulin resistance and inflammation in diabetic rats via irisin/AMPK activation. Sci. Rep..

[73] Collaborators G.B.D.O., Afshin A., Forouzanfar M.H., Reitsma M.B., Sur P., Estep K., Lee A., Marczak L., Mokdad A.H., Moradi-Lakeh M. (2017). Health Effects of Overweight and Obesity in 195 Countries over 25 Years. N. Engl. J. Med..

[74] Dai W., Jiang L. (2019). Dysregulated Mitochondrial Dynamics and Metabolism in Obesity, Diabetes, and Cancer. Front. Endocrinol..

[75] Kugler B.A., Lourie J., Berger N., Lin N., Nguyen P., DosSantos E., Ali A., Sesay A., Rosen H.G., Kalemba B. (2023). Partial skeletal muscle-specific Drp1 knockout enhances insulin sensitivity in diet-induced obese mice, but not in lean mice. Mol. Metab..

[76] Heintz E.C., Dantas W.S., Zunica E.R.M., Belmont K.P., Mey J.T., Axelrod C.L., Kirwan J.P. (2024). Exercise Training Reverses Skeletal Muscle DRP1 Hyperactivation and Improves Respiratory Capacity in Patients with Type 2 Diabetes. Diabetes.

[77] Grosicki G.J., Zepeda C.S., Sundberg C.W. (2022). Single muscle fibre contractile function with ageing. J. Physiol..

[78] Aversa Z., Zhang X., Fielding R.A., Lanza I., LeBrasseur N.K. (2019). The clinical impact and biological mechanisms of skeletal muscle aging. Bone.

[79] Wang D., Sun H., Song G., Yang Y., Zou X., Han P., Li S. (2018). Resveratrol Improves Muscle Atrophy by Modulating Mitochondrial Quality Control in STZ-Induced Diabetic Mice. Mol. Nutr. Food Res..

[80] Esteras N., Abramov A.Y. (2022). Nrf2 as a regulator of mitochondrial function: Energy metabolism and beyond. Free Radic. Biol. Med..

[81] Huang D.D., Fan S.D., Chen X.Y., Yan X.L., Zhang X.Z., Ma B.W., Yu D.Y., Xiao W.Y., Zhuang C.L., Yu Z. (2019). Nrf2 deficiency exacerbates frailty and sarcopenia by impairing skeletal muscle mitochondrial biogenesis and dynamics in an age-dependent manner. Exp. Gerontol..

[82] Hong X., Isern J., Campanario S., Perdiguero E., Ramirez-Pardo I., Segales J., Hernansanz-Agustin P., Curtabbi A., Deryagin O., Pollan A. (2022). Mitochondrial dynamics maintain muscle stem cell regenerative competence throughout adult life by regulating metabolism and mitophagy. Cell Stem Cell.

[83] Kim Y., Triolo M., Hood D.A. (2017). Impact of Aging and Exercise on Mitochondrial Quality Control in Skeletal Muscle. Oxid. Med. Cell Longev..

[84] Zeng Z., Liang J., Wu L., Zhang H., Lv J., Chen N. (2020). Exercise-Induced Autophagy Suppresses Sarcopenia Through Akt/mTOR and Akt/FoxO3a Signal Pathways and AMPK-Mediated Mitochondrial Quality Control. Front. Physiol..

[85] Powers S.K., Radak Z., Ji L.L., Jackson M. (2024). Reactive oxygen species promote endurance exercise-induced adaptations in skeletal muscles. J. Sport. Health Sci..

[86] Schieber M., Chandel N.S. (2014). ROS function in redox signaling and oxidative stress. Curr. Biol..

[87] Wan Y., Liu J., Mai Y., Hong Y., Jia Z., Tian G., Liu Y., Liang H., Liu J. (2024). Current advances and future trends of hormesis in disease. npj Aging.

[88] Iqbal S., Hood D.A. (2014). Oxidative stress-induced mitochondrial fragmentation and movement in skeletal muscle myoblasts. Am. J. Physiol. Cell Physiol..

[89] Yu T., Sheu S.S., Robotham J.L., Yoon Y. (2008). Mitochondrial fission mediates high glucose-induced cell death through elevated production of reactive oxygen species. Cardiovasc. Res..

[90] Pellegrino M.W., Nargund A.M., Haynes C.M. (2013). Signaling the mitochondrial unfolded protein response. Biochim. Biophys. Acta.

[91] Yildirim A.D., Citir M., Dogan A.E., Veli Z., Yildirim Z., Tufanli O., Traynor-Kaplan A., Schultz C., Erbay E. (2022). ER Stress-Induced Sphingosine-1-Phosphate Lyase Phosphorylation Potentiates the Mitochondrial Unfolded Protein Response. J. Lipid Res..

[92] Casas-Martinez J.C., Samali A., McDonagh B. (2024). Redox regulation of UPR signalling and mitochondrial ER contact sites. Cell Mol. Life Sci..

[93] Rovira-Llopis S., Banuls C., Diaz-Morales N., Hernandez-Mijares A., Rocha M., Victor V.M. (2017). Mitochondrial dynamics in type 2 diabetes: Pathophysiological implications. Redox Biol..

[94] Chang X., Niu S., Shang M., Li J., Guo M., Zhang W., Sun Z., Li Y., Zhang R., Shen X. (2023). ROS-Drp1-mediated mitochondria fission contributes to hippocampal HT22 cell apoptosis induced by silver nanoparticles. Redox Biol..

[95] Li J., Zhang S., Li C., Zhang X., Shan Y., Zhang Z., Bo H., Zhang Y. (2024). Endurance exercise-induced histone methylation modification involved in skeletal muscle fiber type transition and mitochondrial biogenesis. Sci. Rep..

[96] Morita M., Prudent J., Basu K., Goyon V., Katsumura S., Hulea L., Pearl D., Siddiqui N., Strack S., McGuirk S. (2017). mTOR Controls Mitochondrial Dynamics and Cell Survival via MTFP1. Mol. Cell.

[97] Bruss M.D., Elliehausen C.J., Clark J.P., Minton D.M., Konopka A.R. (2025). Metformin suppresses the mitochondrial and transcriptional response to exercise, revealing a conserved BCL6B-associated angiogenic program. J. Appl. Physiol..

